# Long-Term Mortality and Morbidity Related to Congestive Heart Failure with Reduced Ejection Fraction (CHFrEF) in Palestinian Patients Maintained on Submaximal Sacubitril/Valsartan Doses: A Pilot Study

**DOI:** 10.1155/2021/1829873

**Published:** 2021-12-28

**Authors:** Raed Aqel, Tareq Z. Alzughayyar, Sadi A. Abukhalaf, Rami A. Misk, Jihad Samer Zalloum

**Affiliations:** ^1^National Center for Diabetes, Endocrinology and Genetics, Jordan; ^2^College of Medicine and Health Sciences, Palestine Polytechnic University, Hebron, State of Palestine; ^3^Al-Quds University, Faculty of Medicine, Jerusalem, State of Palestine

## Abstract

**Background:**

The efficacy of sacubitril/valsartan, a newly introduced combination drug for heart failure with reduced ejection fraction (HFrEF), was demonstrated in the PARADIGM-HF trial conducted in Western countries. However, these findings need to be verified in the Middle Eastern context, where patients may exhibit a different response due to different environmental and racial factors.

**Objectives:**

The goal of this study was to evaluate the efficacy of submaximal sacubitril/valsartan doses in terms of improving the disease symptoms, as measured by the New York Heart Association (NYHA) classification and left ventricular ejection fraction (LVEF) percentage, as well as establish long-term morbidity and mortality associated with HFrEF among Palestinian patients administered target doses of an angiotensin-converting enzyme inhibitors (ACEI) or angiotensin II receptor blockers (ARBs). *Material and Methods*. This study involved a retrospective review of charts related to patients with HFrEF maintained on sacubitril/valsartan and was conducted in a referral cardiology clinic in Palestine. The inclusion criteria were age 18+, HFrEF diagnosis, sacubitril/valsartan usage for at least six months during the period between January 1, 2016, and June 30, 2019, and LVEF < 40%. The exclusion criteria included LVEF ≥ 40% and drug administration duration < 6 months. The collected data included NYHA class, as well as LVEF, serum sodium (Na), potassium (K), serum creatinine (Cr), and blood urea nitrogen (BUN) levels and the mortality rate before and after the minimum treatment duration. IBM SPSS STATISTICS for Windows, version 20.0, Armonk, NY: IBM Corp. IBM Corp., released 2012, was used for data analysis, whereby *T* score was calculated for comparisons between numerical groups, and *p* < 0.05 was considered statistically significant.

**Results:**

The initial study sample comprised of 205 consecutive patients with HFrEF maintained on sacubitril/valsartan for at least six months from January 1, 2016, to June 30, 2019. Three patients were excluded due to attrition, along with further 12 patients with LVEF ≥ 40% (based on the PARADIGM-HF trial criteria). Throughout the treatment period, most patients showed escalating improvement in terms of the LVEF and NYHA classification, as LVEF = 29.8% and NYHA = 3 were obtained on average before initiating sacubitril/valsartan, compared to 41% and 1.7, respectively, after 6-month treatment (*p* = 0.0003 and 0.046, respectively). These improvements in LVEF and NYHA class were noted across all sacubitril/valsartan doses (50−400 mg). However, 23 patients (12%) died while undergoing sacubitril/valsartan treatment.

**Conclusion:**

A significant long-term reduction in the mortality and morbidity rates was observed in Palestinian patients with HFrEF maintained on submaximal doses of sacubitril/valsartan.

## 1. Introduction

Congestive heart failure (CHF) is considered a costly ailment due to its significant economic burden and higher morbidity and mortality rates across the globe than those arising from most other illnesses, including cancers [1]. Although the same is true for the Middle East, apart from a few scattered reports, the exact statistics regarding prevalence and long-term morbidity and mortality secondary to heart failure with reduced ejection fraction (HFrEF) are lacking [2].

Recently, sacubitril/valsartan was approved as a treatment for HFrEF patients as its superior efficacy was demonstrated in the PARADIGM-HF trial conducted in 2015 in terms of reducing mortality and morbidity compared to traditional treatments involving maximal doses of ACE inhibitors [3]. Sacubitril/valsartan is a combination medication that contains an angiotensin II receptor blocker (ARB) and a neprilysin inhibitor. As neprilysin is responsible for the degradation of atrial and brain natriuretic peptides, the cardiovascular and renal effects of sacubitril's active metabolite (LBQ657) in heart failure are attributed to the increased levels of peptides that are degraded by neprilysin (e.g., atrial natriuretic peptide), whereby its administration results in increased natriuresis and urine cyclic guanosine monophosphate (cGMP) and decreased plasma midregional pro atrial natriuretic peptide (MR-proANP) and N-terminal pro b-type natriuretic peptide (NT-proBNP). On the other hand, the angiotensin II receptor type I inhibitor (ACEI), valsartan decreases blood pressure and blocks the vasoconstriction and aldosterone-secreting effects of angiotensin II [4–6]. The evidence yielded by the PARADIGM-HF trial, and the clinical experience in the Middle East (the Gulf region in particular) indicates that sacubitril/valsartan is associated with reduced short-term mortality and hospitalization rates in CHF patients irrespective of ACE inhibitor administration in target doses [2, 3]. Consequently, sacubitril/valsartan is currently recommended for patients with symptomatic HFrEF classified as New York Heart Association (NYHA) Class II or III [7].

In our previous publication, we demonstrated LVEF improvements in Palestinian patients with HFrEF who took sacubitril/valsartan regardless of the disease etiology [6]. Since no data regarding long-term mortality and morbidity related to HFrEF in the Middle East is presently available, this novel study and the long-term evidence based on following up patients with HFrEF maintained on variable submaximal doses of sacubitril/valsartan for up to four years are immensely valuable for this region.

## 2. Material and Methods

This single-center retrospective chart review study was conducted in a referral cardiologist clinic in Palestine. The study commenced on December 31, 2019, and the data regarding all patients who were prescribed sacubitril/valsartan between January 1, 2016, and June 30, 2019, was collected. All patients agreed voluntarily for their data to be used as a part of this study and signed an informed consent form after being informed of the study protocols and aims.

**Figure 1:**
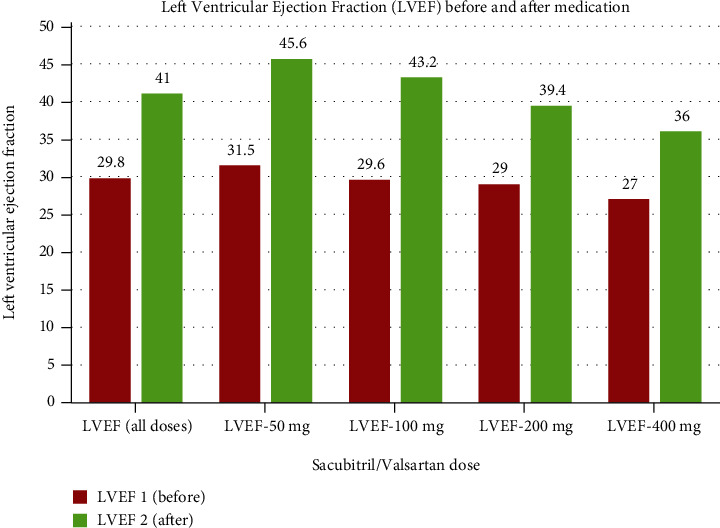
Graphs show changes in the LVEF in relation to different accumulative medication doses. For patients on 50 mg of sacubitril/valsartan daily, an average of LVEF1 = 29.8 and LVEF2 = 41 was achieved. For patients on 100 mg daily, an average of LVEF1 = 31.4 and LVEF2 = 43.8 was achieved. Finally, for those receiving 200 mg daily, an average of LVEF1 = 28.3 and LVEF2 = 38.5 was obtained. In all cases, the *p* value was statistically significant (i.e., below the 0.05 cut-off).

**Figure 2:**
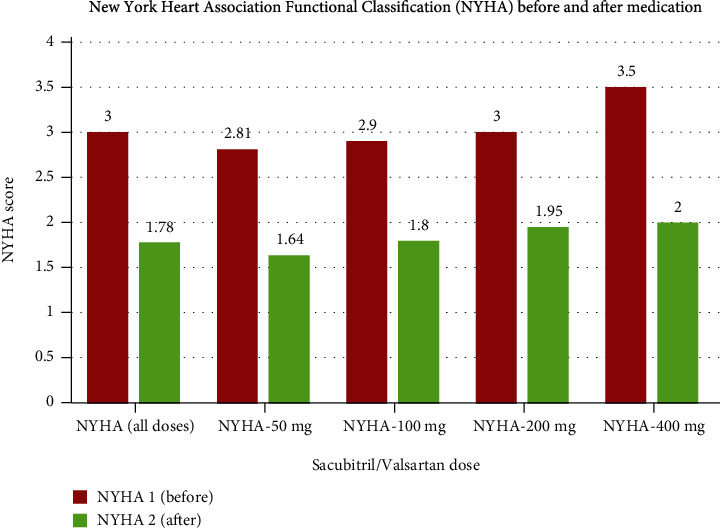
Graphs show the changes in the NYHA classification with different medication doses. For patients on 50 mg, 100 mg, and 200 mg of sacubitril/valsartan daily, the average values were NYHA1 = 2.9 and NYHA2 = 1.64, NYHA1 = 3 and NYHA2 = 1.8, and NYHA1 = 3.1 and NYHA2 = 1.95, respectively. In all cases, the *p* value was statistically significant (i.e., below the 0.05 cut-off).

The inclusion criteria were age 18+, HFrEF diagnosis, sacubitril/valsartan usage for at least six months during the period between January 1, 2016, and June 30, 2019, and LVEF < 40%. The exclusion criteria included LVEF ≥ 40% and drug administration duration < 6 months. We collected the data retrospectively through a paper-based and electronic chart review. As a result, the follow-up period ranged from 4 years (patients who started taking sacubitril/valsartan on January 1, 2016) to 6 months (patients who started taking the medication on June 30, 2019). The collected data included NYHA class, as well as LVEF, serum sodium (Na), potassium (K), serum creatinine (Cr), and blood urea nitrogen (BUN) levels, in addition to the mortality rate before and after the minimum treatment duration. For patients for whom multiple NYHA and LVEF were recorded, the last recorded value before commencing the treatment served as the control value. The most recent record after treatment completion served as the result value in the descriptive analysis of demographic variables, as summarized in Tables 1 and 2 and shown in Figures 1 and 2. All analyses were performed using the IBM SPSS Statistics for Windows, version 20.0, Armonk, NY: IBM Corp. IBM Corp., released 2012, commercial software and included a *T* score for comparison between numerical groups, with *p* < 0.05 signifying statistically significant findings.

## 3. Results

The chart review revealed that 205 patients with HFrEF were maintained on the sacubitril/valsartan medication during the period from January 1, 2016, to June 30, 2019. However, 12 patients who had pretreatment LVEF > 40% and three individuals that did not attend all follow-up appointments were excluded. Thus, the final sample comprised of 190 patients who had documented charts for at least six months (up to four years).

The most commonly reported HFrEF etiology was ischemic heart disease (65.2%), followed by atrial fibrillation (15.2%), whereas hypertension (119 patients, 62.6% of the sample) and diabetes mellitus (95, 50%) were the most prevalent comorbidities, as shown in Table 1.

Chart review further revealed that 177 patients (93.1%) were prescribed ACEI or ARBS, though only 20 patients (10.5%) were on the target doses of either medication before switching to sacubitril/valsartan.

Although no specific protocol was used to initiate sacubitril/valsartan administration, patients who presented with LVEF < 40% and/or NYHA Class II or greater were prescribed the drug, whereby the initial doses were determined by the treating physician while considering tolerability and the patients' ability to pay for the medication.

**Figure 3:**
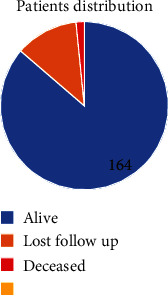
June 30, 2019, follow-up of 190 patients who were maintained on sacubitril/valsartan for at least six months (up to four years), indicating that 164 were still alive, 23 had died, and three did not attend the follow-up appointment.

**Figure 4:**
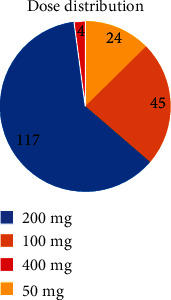
The number of patients who were maintained on different cumulative sacubitril/valsartan doses: 24 patients received 50 mg, 45 patients 100 mg, 117 patients 200 mg, and 4 patients 400 mg daily.

As shown in Figures 3 and 4, 24 patients (12.6%) were maintained on 50 mg once daily (24/26 mg), 45 patients (23.7%) were on 50 mg twice daily (24/26 mg), 66 patients (34.7%) were on 100 mg twice daily, 51 patients (26.8%) were on 200 mg once daily (97/103 mg), and only 4 patients (2%) were prescribed the maximum dose of 200 mg twice daily (97/103 mg). During the observation period, 21 patients (11%) stopped taking the medication.

Analyses further revealed that 12 patients (6.3%) had LVEF in the 40−45% range and were thus excluded from the study based on the PARADIGM-HF criteria. Among the remaining patients, 98 (47.8%) had LVEF in the 30−40% range, and in 92 patients (44.8%), LVEF < 30% was noted. In terms of the NYHA class, 31 patients (16.3%) were in Class II, 106 patients (55.7%) in Class III, and 53 (27.9%) in Class IV. Of the 190 individuals whose records were included in the review, 23 (12% accumulative mortality rate) had died while taking sacubitril/valsartan, with terminal heart failure complications and cardiac arrest cited as the causes of death.

As shown in Figures 1 and 2, most patients exhibited escalating improvement in terms of the LVEF and NYHA class over the sacubitril/valsartan treatment period regardless of the dose administered. The mean LVEF value before sacubitril/valsartan initiation was 29.8%, increasing to 41% after at least six months of treatment (*p* = 0.003) (Figure 1). Similarly, regardless of the prescribed dose, there was a consistent improvement in the NYHA classification (Figure 4), as the mean values of NYHA class before and after treatment were 3 and 1.78 (*p* = 0.046), respectively, as shown in Table 2.

For the sample as a whole, decreased mean serum sodium levels, along with increased mean serum potassium and mean renal function levels (creatinine and blood urea nitrogen), were recorded. The mean calculated creatinine values before and after treatment were 1.13 mg/dL and 1.26 mg/dL, respectively, whereas 4.1 mmol/L and 4.4 mmol/L were obtained for potassium and 137.5 mmol/L and 136.34 mmol/L for sodium, as shown in Table 2.

Although sacubitril/valsartan treatment discontinuation (11%) was largely due to the high financial burden, gastrointestinal symptoms, itching, and worsening kidney function (only one patient) were also noted in several cases.

In patients that did not take sacubitril/valsartan as prescribed (largely due to inability to pay), LVEF and the NYHA class exhibited improvements and deterioration corresponding to medication intake and abstinence.

## 4. Discussion

HFrEF is considered a global epidemic, as it is associated with nearly 40% and 50% 4-year and 5-year mortality rates, respectively, despite treatment [1]. HFrEF also imposes a high economic burden in most countries, as it is responsible for more days off work than all other diseases combined, as well as the highest expenses related to recurrent hospitalizations and devices required [8]. Recently, Zhao et al. conducted a meta-analysis and found that the earlier sacubitril/valsartan version was superior to ACEI in its ability to reduce the risk of significant cardiac adverse events and increase left ventricular ejection fraction [9]. Thus, the new medication (sacubitril/valsartan, LCZ 696, Entresto) was introduced and is currently recommended by most guidelines as an alternative to classic ACE inhibitors due to a 20% improvement in morbidity and mortality [3].

However, owing to the different lifestyle, racial, and environmental characteristics, these rates might not be attainable in the Middle East and the Arab countries. As no database on the long-term mortality and morbidity rates related to heart failure presently exists, most practitioners have to rely on the few short-term mortality reports related to the Gulf area [10].

For example, limited data on the Palestinian Arab communities in the West Bank shows that HFrEF could be more common than in Western countries [8, 11], likely due to the health system deficiencies and thus the inability to address the risk factors such as coronary artery disease (CAD), hypertension (HTN), diabetes mellitus (DM), smoking, and hyperlipidemia, which in turn increase the prevalence of heart failure [10, 11]. However, in a previous study involving Palestinian patients with HFrEF, a highly positive response to sacubitril/valsartan was noted in terms of improving ejection fraction and NYHA classification [11], as well as a decrease in the complication rates and related expenses [8]. Our study showed favorable outcome in terms of lower mortality and morbidity relative to international rates, despite submaximal doses of sacubitril/valsartan, e.g., (50 mg or 100 mg total daily dose) and breaks in treatment in some patients (due to financial hardship). These findings, while highly informative, are not meant to encourage healthcare providers to prescribe submaximal doses of any medication but rather to indicate that improvements can be attained even with submaximal doses.

Finally, despite technical and economic challenges such as patients' partial compliance due to drug unaffordability, scarcity of national database, and small sample size, this study has made significant contributions to the extant literature. In particular, as it is the first of its kind in Palestine, as it sheds light on the needs of HFrEF patients and the long-term morbidity and mortality rates in this population. We thus hope that this article will motivate other researchers to study HFrEF and the different benefits of sacubitril/valsartan in patients of Arab ethnicity, such as its impact on cholesterol and HbA1c.

## 5. Conclusion

Sacubitril/valsartan is a novel revolutionary drug for patients suffering from HFrEF. Our analyses indicate that it yields major benefits in improving the long-term mortality and morbidity rates of Palestinian patients regardless of the prescribed dose. Thus, we hope that this work will serve as a starting point for further investigations into HFrEF and sacubitril/valsartan in the Middle East.

## 6. Limitations

The limitations of this study are as follows: a retrospective chart review study, a small sample size (only 190 patient records were analyzed), and no national long-term morbidity and mortality data are available for comparison.

## Figures and Tables

**Table 1:** Patient demographics and initial findings.

Patients' background	Number of patients
Ischemia	124
DM	95
HTN	119
AICD	18
CRTD	8
AF	29
Smoking	60

DM: diabetes mellitus; HTN: hypertension; AICD: automatic implantable cardioverter-defibrillator; CRTD: cardiac resynchronization therapy defibrillator; AF: atrial fibrillation.

**(a):** 

Variable	Value 1 (before medication)	Value 2 (after medication)
Left ventricular ejection fraction	29.8%	41%
New York Heart Association	3	1.78
Creatinine	1.13 mg/dL	1.26 mg/dL
Blood urea nitrogen	25 mg/dL	25.7 mg/dL
Sodium	137.5 mmol/L	136.3 mmol/L
Potassium	4.1 mmol/L	4.4 mmol/L

**(b):** 

Alive	167
Deceased	23
No follow-up	3

## Data Availability

The data is available to review at request.
